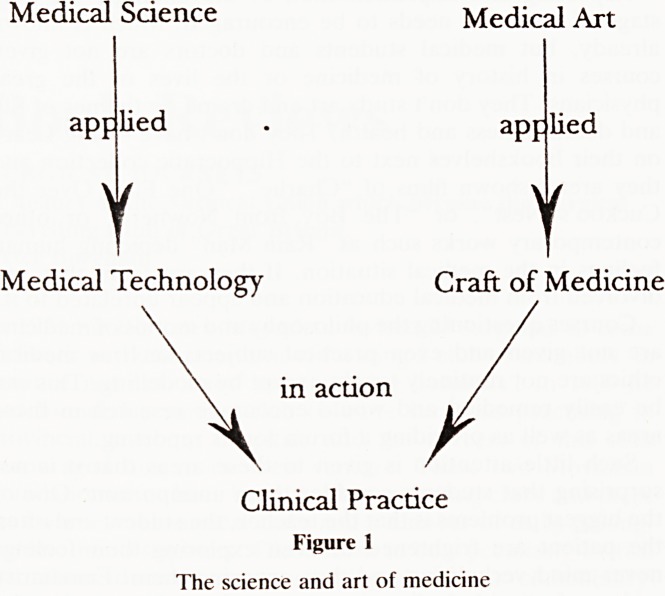# Teaching and Research of the Art of Medicine

**Published:** 1990-12

**Authors:** Andrew J. Cave

**Affiliations:** Research Fellow in General Practice, General Practice Unit, University of Bristol General Practitioner, Highbridge, Somerset


### Teaching and Research of the Art of Medicine

**Andrew J. Cave MB, ChB, MClSc, MRCGP**
Research Fellow in General Practice,
General Practice Unit, University of Bristol

General Practitioner, Highbridge, Somerset

Contemporary medicine is claimed to be both a science and an art. However, throughout this century the scientific emphasis has been predominant.

Technology has been considered as being applied science and craft as being applied art. In medicine we may consider clinical practice as both the applied science and also the applied art of medicine since it combines both the technology and the craft. (Fig. 1). Teaching or researching the craft of medicine without the art would be the equivalent of teaching the technology without the basic sciences, but this is often what happens in the medical curriculum. Some G.P. trainees when being taught the clinical craft find they are doing so in a vacuum because they have never been taught the art.

**Figure 1**

The science and art of medicine

It has to be acknowledged that in medicine it is more difficult to teach the craft than the technology as it is probably harder to learn the art than the science. The good student acquires much of his understanding of the art by observing his teacher practising the craft rather than by direct teaching. For this reason Byrne[1] in his book "Learning to Care" strongly advocated the one to one attachment for learning general practice.

One of my own trainees who, in assessing her learning during the previous six months with me, felt she had not learned much "new medicine", but rather what she called "the intangibles" of caring for and interacting with patients. She felt her time had been very fruitfully spent.

The science of medicine, that part based on theories verifiable by experiment, can be researched by the scientific methods of epidemiology or by laboratory experiment. The art of medicine has been much less researched than the science. Indeed of ten reference books on medical research reviewed not one mentioned the art or the methods of researching it[2]. The training of doctors in this century has been almost exclusively in the scientific method.

Correspondence to: Dr. A. J. Cave, The Surgery, Market Street, Highbridge, Somerset TA8 3ED

Medical students are not expected to have a classical or philosophical education before embarking on their basic-science course. Latin, which was compulsory thirty years ago as an entry qualification for medical school is now no longer required. Few British medical schools admit first M.B. or "Pre-med" students. This used to be the accepted method for students of the humanities to enter medicine. There are only a few medical schools that admit mature students and others with a non-scientific background.

The recent resurgence of interest in people as individuals and the shift in society's thinking away from technology seems an ideal time to re-emphasise the art of medicine. Research now could expand our understanding of this major component of our discipline and help us define it. General practice is an ideal field in which to do such research, being as it is on the very fringe of technological medicine and so dependent on the art.

Polanyi[3] says "an art which cannot be specified in detail cannot be transmitted by prescription—" If we hope to teach the art of general practice in this way we must attempt urgently, by research, to specify its intangibles because the technological era has lasted 100 years and "an art which has fallen into disuse for the period of a generation is—" according to Polanyi, "altogether lost"[4].

There is little doubt that general practice is an "immature" discipline as defined by Ravetz,[5] and exposed by McWhinney[6]. As in all immature disciplines, doctors will have to be trained in several different methods of research.

To research the art of medicine it will be necessary to use other methods besides the scientific method, and we can look to the humanities and art for examples of these—"a literate art would naturally be based partly on the 'history' of its objects and be informed to some extent by a philosophy of its principles"[7].

The basic princples of inquiry by history and philosophy are; observation and faithful comprehensive accounts in *history*; and reflection, observation and coherence of argument in *philosophy*. Both are exemplified by curiosity, observation and honesty. Historical method may help us to research the art as it is followed and the philosophical method help us research the question "are we going the right way?" The knowledge of science is expressed as facts, that of philosophy as aphorisms. One is determined deductively the other inductively, but both are just as valid as truths.

Quoting Ravetz again[8] "the recognition of 'art' as a category distinct from inquiry could also contribute to a better self-understanding of immature fields". An artist's knowledge is subjective and personal and art communicates feelings[9]. The artist is concerned with the religious, moral, existential meaning of experience and his symbols are metaphorical.

In their book "Methods of Research" Good and Scates[10] take an overall view of research and they define the steps in research of any subject, but of history in particular. Matczak in "Research and Composition in Philosophy" lists the steps and sources also[11]. The two lists are very similar and are compared in abbreviated form in Table 1.

Matczak also includes in his process of research,[12]

inductive reasoning
use of hypotheses
causation
historical perspective and
the principle of synthesis

Before it can be researched, the authors all agree on the starting point, a definition of "the art of medicine".

According to Ravetz,[13] Aristotle looked on an art as "the set of principles defining the methods of any class of tasks".

**Table 1**

**Steps in research method**

| *Good and Scates (preface q.v.)* | *Matczak (Chpter II)* | |
|---|---|---|
| Formation of the problem | Define the subject | |
| Literature Survey | Gathering material | Heuristic |
| Selection and use of appropriate method | | Stage |
| Analysis | Analysis | Hermenentic |
| Interpretation | Criticism | Stage |
| Reporting and implementation | The composition | |

Ravetz himself looking at a discipline and wishing to identify its art says[14] "the successful 'arts' could be recognised as the most genuine tested experience of the field and studied and developed as such".

True art has been defined by Brennan as "an experience which is conveyed between one person and another"[15]. The art of medicine has been defined by McWhinney as "the process by which we understand the patient as a person"[16] or as "the physician—patient relationship"[17].

From these, a definition of the art of medicine to be researched could be, "what is involved in understanding and experiencing the doctor, the patient and the relationship as expressed between the established physician and his real patient in generally acknowledged normal practice".

So in their research, general practitioners may use methods that are personal and even subjective, that study feelings, the religious, moral and existential meaning of experience and use the interpretation of metaphor. They will research the unique meaning of the illness to this particular patient, or the language of feelings, or human relationships or the personal experience, both past and present, of the physician.

How then will they gather material for researching the art of medicine? Following the outline by Good and Scates they will choose the appropriate method. Some aspects may be best researched scientifically by the behavioural sciences, but they will only be part of the field and general practice must avoid falling into the trap of only researching those aspects available to this method. The historical philosophical and artistic methods can also be used to investigate the art.

Medical students could study the writings of the great masters of the art, Hippocrates, Galen, Harvey and more recently Jenner, MacKenzie and Pickles. They could also learn a great deal from studying the men themselves, from reading their biographies and from research into the social history in which they lived and practised their art. The history of medicine itself and the development of the art could be included here.

Research of works of art related to medicine is most important. Study of literary works, paintings and drama by artists (such as Tolstoy) with insight into the subject of medical practice is rewarding to anyone. The works of doctors who are artists of any medium could be particularly studied and even compared with works by non-medical men such as lawyers. Medical students and doctors could be asked to express their feelings in art, poetry or writing during or after a consultation. This could be done with doctors of different ages and experience and with cases of different levels of emotional content.

The art of the Shaman, faith healer and outright quack could be researched both from their writings[18] and by direct observations both descriptively and if necessary scientifically by experiment. This latter method is already used to study the placebo effect and could be extended into a study of the art of the healer itself.

Research of the feelings and responses of doctors and patients to their encounters as in Balint-type groups would be most informative particularly when applied to those cases which require most art e.g. the sorrowful or the dying for whom science can offer so little. This form of research could be very meaningful even in the form of individual case histories without any numbers to give "statistical significance".

The behavioural sciences already offer some assistance, and could be used more in researching interviewing skills, personal relationships and the language of feelings. Tehir findings could be extended to, or re-examined in, the doctor—patient relationship. Once again there is a scientific content but the artist uses scientific knowledge quite authentically to improve his materials and tools.

The interpretation, analysis and synthesis of all the accumulated knowledge will of course, be open to debate and discussion "ad infinitum" just as are the aphorisms of history and philosophy. Each researcher will make his own subjective interpretation particularly of the artistic research, and rightly so. It may be some time before a general truth can be discerned or before the man of wisdom appears who can discern and synthesise it. The art of medicine has been practised and reflected on for centuries. By research we can at least add to the knowledge of the art for future practitioners.

Reporting and implementation of the findings, the next stage of research, needs to be encouraged. Much is known already, but medical students and doctors are not given courses in history of medicine or the lives of the great physicians. They don't study art and drama on themes of life and death, illness and health. They don't have "King Lear" on their bookshelves next to the Hippocratic collection and they aren't shown films of "Charlie", "One Flew Over the Cuckoo's Nest", or "The Boy from Nowhere" or other contemporary works such as "Rain Man" depicting human feelings in the medical situation. If they are seen, they are divorced from medical education and appear unrelated to it.

Courses questioning the philosophy and morals of medicine are not given and even practical subjects such as medical ethics are not routinely taught except by modelling. This can be easily remedied and would encourage research in these areas as well as providing a forum for its reporting.

Such little attention is given to these areas that it is not surprising that students consider them unimportant. One of the biggest problems is that the teacher, the student and often the patient are frightened of even exploring their feelings never mind verbalising and thus exposing them. Familiarity and comfort with feelings can be taught and researched by experimental courses and Balint-groups, and by the example of teachers researching their own feelings. The concept of "subjective intelligence" propounded by Novak[19] could be re-introduced to the medical mind.

We can anticipate problems with our research both from within and from without the profession. From within, the problems include observing, recording and interpreting subjective data, the unspoken language of feelings, and overcoming our own and our colleagues' feelings. We have also to surmount the loss of respect for pure learning and the reluctance of doctors to undertake teaching and research.

There will also be problems with the internal organisation of general practice, co-operation of researchers and definition of direction as well as dispute of the methodology described here.

General practice is the obvious front line for the research of the art since this is the field where it can be, and is, most practised. However, general practice is an immature discipline, and like all immature disciplines[20] general practice may be tempted, due to pressures from without, to declare its maturity too soon in order to achieve recognition, thus closing the doors on possible directions of growth. On the other hand it might rush into "research for its own sake" since the volume of published research provides an "opportunity for an expansion of the institutional apparatus including an academic base"[21]. The danger is hypertrophy, with large academic departments teaching minimal content and producing poor or meaningless research. However some people may feel that if general practice admits too loudly to the immaturity of its discipline it will not receive facilities, funds or academic openings for research to continue.

These problems must be overcome because general practice, indeed the whole of medicine, will stay an immature field so long as "the absence of a body of appropriate methods of inquiry nullifies (our) efforts"[22]. It is therefore most important for the future growth to maturity of medicine that we address the problem of methodology for researching the ever developing and varied art of medicine.

### REFERENCES

1. BRYNE, P. S., LONG, B. E. L. (1973) Learning to Care Person to Person. Edinburgh: Churchill Livingstone.
2. A continuous sample of the reference section on medical research in U.W.O. Science Library. University of Western Ontario, London. Ontario.
3. POLANYI. (1958) Personal Knowledge: Towards a Post-critical Philosophy. Chapter 4.3, page 53. Chicago: University of Chicago Press.
4. ibid. p. 53.
5. RAVETZ, J. (1973) Scientific Knowledge and its Social Problems. p. 365. New York: Oxford University Press.
6. McWHINNEY, I. R. (1978) Family Medicine as a Science. *Journal of Family Practice*. Volume 7, No. 1, p.56.
7. RAVETZ, J. *op. cit.* p. 373.
8. RAVETZ, J. *op. cit.* p. 373.
9. McWHINNEY, I. R. (1978) Medical Knowledge and the Rise of Technology. *Journal of Medicine and Philiosophy*. Volume 3, No. 4, p. 298.
10. GOOD, C. V. and SCATES, D. E. (1954) Methods of Research. New York: Appleton-Century-Crofts.
11. MATCZAK, S. A. (1971) Research and Composition in Philiosophy. Louvain: Editions Nauwelaerts.
12. MATCZAK, S. A. ibid.
13. RAVETZ, J. op. cit. p. 373 and p. 374.
14. RAVETZ, J. op. cit. p. 373 and p. 374.
15. BRENNAN, M. (1982) Introduction to Whole Person Medicine Course-work guidelines. *Dept. of Family Medicine,* University of Western Ontario.
16. McWHINNEY, I.R. (1976) Medicine as an Art Form. *Canadian Medical Association Journal.* Volume 114. p. 98.
18. JAMESON, E. (1961) The Natural History of Quackery. London: Michael Joseph.
19. NOVAK, M. The Liberation of Imagination (The place of intelligent subjectivity in health care education) p. 95.
20. RAVETZ, J. op. cit. p. 377–378.
21. ibid. p. 385.
22. ibid. p. 369.

## Figures and Tables

**Figure 1 f1:**